# Evolution of Free Amino Acids, Histamine and Volatile Compounds in the Italian Anchovies (*Engraulis encrasicolus* L.) Sauce at Different Ripening Times

**DOI:** 10.3390/foods12010126

**Published:** 2022-12-27

**Authors:** Onofrio Corona, Luciano Cinquanta, Caterina Li Citra, Francesca Mazza, Vincenzo Ferrantelli, Gaetano Cammilleri, Emanuele Marconi, Francesca Cuomo, Maria Cristina Messia

**Affiliations:** 1 Dipartimento Scienze Agrarie, Alimentari e Forestali, Università di Palermo Viale delle Scienze, Ed. 4, 90128 Palermo, Italy; 2 Istituto Zooprofilattico Sperimentale della Sicilia, Via Gino Marinuzzi 3, 90129 Palermo, Italy; 3 Dipartimento di Agricoltura, Ambiente e Alimenti, Università degli Studi del Molise (DiAAA), Via de Sanctis, 86100 Campobasso, Italy; 4 Facoltà Dipartimentale STUA, Università Campus Bio-Medico di Roma, Via A. del Portillo 21, 00128 Roma, Italy

**Keywords:** fish sauce, food safety, nutritional quality, biogenic amines, fermented seasoning

## Abstract

In this study, the evolution of the safety, nutritional, and volatile profile of a traditional Italian anchovy sauce with protected designation of origin (PDO), called “colatura di alici di Cetara”, is investigated after 12, 24, and 48 months of aging in wooden barrels. Some physicochemical parameters, free amino acids, volatile compounds, and histamine contents were evaluated during the aging of the samples. Glutamate, which together with aspartate is responsible for the umami taste, was the predominant free amino acid in the tested fish sauce, with a significant increase during the 48 months of maturation. The total amino acid content of the anchovy sauce increased from 24 to 48 months of aging. The histamine content decreased significantly from 12 to 48 months of ripening. This point is particularly interesting for the sauce safety and confirms the importance of the maturation time of at least 9 months reported in the disciplinary of production. A total of 44 volatile compounds were found in the anchovy sauce samples, of which the largest class was acids, mainly isovaleric acid. The results show that prolonged maturation improves the safety, nutritional, and volatile components of the seasoning “colatura di alici”.

## 1. Introduction

Fish sauce is a naturally valuable, amber, and widely used fermented seasoning [1]. There is a wide variety of fish sauce-producing countries around the world; the leading producer is Thailand. Natural fermentation is considered a traditional phase in the production of fish sauces, the most famous of which, according to Pliny the Elder (AD 23–79), is known as Garum, “liquor exquisitus”, the most common condiment used in the ancient Roman Empire to improve the flavor of widely consumed dishes [2]. Under the synergistic action of halophilic microorganisms and enzymes, fish proteins and fats are degraded through various biochemical metabolic pathways to form the unique flavor of fish sauce, which is influenced by the microbial population composition and different environmental conditions (e.g., temperature, time, etc.) [3,4]. The manufacturing process essentially consisted of a dry salting process, in which the Romans placed small whole fish or the innards of large fish in a container with salt in a specific ratio, possibly along with other ingredients. The ancient technique of dry salting is very similar to modern fish sauce production in Southeast Asia, Japan, China [5,6], and Europe. On the Amalfi Coast (Italy), since 1807, anchovies caught are gutted, beheaded by hand, and placed in wooden vats in alternating layers separated by sea salt. Once the layers are ready, a wooden disk with weights is placed on top of the vats, and the aging process starts. The clear liquid sauce, obtained from pressed anchovies matured in wooden vats under salt for at least 9 months, obtained the status of protected designation of origin (PDO) by the European Union in 2020, with the name “*colatura di alici di Cetara*” [7]. 

An important marker for assessing the quality of fish is the aroma, which is formed by the degradation of lipids and proteins through various metabolic pathways under the action of halophilic microorganisms and enzymes [3]. The evolution of volatile components, such as alcohols, ketones, and aldehydes, depends mainly on lipolysis and fatty acid oxidation during fermentation [8,9]. In addition, biogenic amines, such as histamines, are formed during the rapid decomposition of fish proteins by the enzymes produced by bacterial growth. Histamine can cause toxicological symptoms, such as pseudo-allergic reactions, and its content in anchovy sauces is a quality indicator. The upper limit of histamine content in foods (400 mg/kg) is set in the European Regulation 1019/2013 [10] to ensure consumer safety. Some halophilic bacteria can play the role of histamine suppressors during long fermentation, through the activity of histamine dehydrogenase or histamine oxidase. It is also known that the *colatura di alici* sauce (POD) becomes more valuable the longer the maturation period, as its sensory and nutritional properties improve due to the proteolysis progress and the formation of volatile compounds. This would confirm what has been reported for ancient Garum aged up to 12 months and for some of the fish sauces from the Far East aged up to 24 months. In light of this, sauce samples of colatura di alici were collected and analyzed at different maturation periods (12, 24, and 48 months), and the effectiveness of microbial activities on nutritional and safety quality improvement has been assessed.

## 2. Materials and Methods

### 2.1. Samples and Process Description

The samples of anchovy sauce were from the producer (Rossi s.n.c.) in Cetara (SA, Italy), who follows the PDO discipline of *colatura di alici di Cetara* [7]. In short, the fish are gutted and beheaded by hand and then placed in wooden barrels called terzigni (one-third of a barrel). Anchovies and sea salt are arranged in alternating layers, with a minimum weight ratio of anchovies to salt of 2:1. The barrels are then covered with a wooden lid, on which pressure is applied to remove trapped air. The anchovies are matured at a temperature of 18–25 °C.

Samples were taken from different wooden barrels after 12, 24, and 48 months of maturation. The barrels chosen were representative of the anchovies’ sauces at three stages of maturation in this research, which took place on a farm with 50 barrels. More precisely, sampling covered 30% of the barrels with 12 months of maturation and 60% with 24 and 48 maturation months, as each sample was composed by mixing the aliquots of liquid spilled by three different barrels for each maturation stage. The liquid was collected from the bottom of the barrels through a hole drilled with a thin screw. Two other samples of commercial anchovy sauce were used as references—one from Cetara and the other from Sicily (Sciacca, AG, Italy)—and were purchased from local supermarkets. All samples had a salt content of 20% (*w*:*w*). The sample coding used is as follows:

C1, commercial sample (Sicily region, Sciacca (AG), Italy)

C2, commercial sample (Campania region, Cetara (SA), Italy)

R1, 12-month ripened sample (PDO Rossi Company, Campania region, Cetara (SA), Italy)

R2, 24-month ripened sample (PDO Rossi Company, Campania region, Cetara (SA), Italy)

R3, 48-month ripened sample (PDO Rossi Company, Campania region, Cetara (SA), Italy).

Each sample was analyzed in triplicate.

### 2.2. Physical and Chemical Analysis

The determination of the moisture content (%) was carried out by drying samples in an oven at 105 °C for 16–18 h. The pH was measured using a pH meter (Basic 20, Crison Instruments SA, Alella, Barcelona, Spain).

Color measurements were performed using a Konica Minolta chroma meter CR-400 (Konica Minolta Sensing Singapore Pte Ltd., Osaka, Japan). L*, a*, b*, chroma (C), and hue angle (H°) values were considered. 

Water activity, *a*w, was measured at 25 °C as vapor pressure through the dew point using an Aqualab CX-2 instrument (Decagon Devices, Pullman, WA, USA). 

### 2.3. Free Amino Acids Determination

An analysis of free amino acids was performed using the ICS6000 chromatographic system (Thermo Fisher Scientific S.p.A, Milano, Italy). Samples of fish sauce were diluted 1:2000 with ultra-pure water before the injection in the chromatographic apparatus. The separation was carried out at 30 °C with an Aminopac PA10 analytical column (250 × 2 mm, 8.5 μm particle size) (Thermo Fisher Scientific S.p.A, Milano, Italy), using NaOH 250 mM and sodium acetate 1 M as eluent water, and a time/potential waveform according to the conditions shown in Table 1.

### 2.4. Histamine Determination

Histamine analysis was carried out according to the method proposed by Cinquina, et al. [11]. Briefly, fish sauce samples of about 10 g were transferred to centrifuge tubes and homogenized with 10 mL of 6% perchloric acid. The homogenates were centrifuged (Sigma 3–18 K, Sartorius, Germany) at 10,000× *g* and 4 °C for 10 min and filtered through Whatman paper No.1. Standard solutions of histamine dihydrochloride were also prepared to obtain the calibration curve. An HPLC analysis was performed with an Agilent 1290 chromatography system equipped with a UV/DAD detector set at 210 nm (Agilent Technologies Inc., Santa Clara, CA, USA). The chromatographic column used was a Supelcosil LC-ABZ C18, 5 μm, 150 × 4.6 mm. Phosphate buffer pH 6.9 (85%) and acetonitrile (15%) were used in the isocratic elution system, with a flow rate of 1.2 mL/min.

### 2.5. Analysis of Volatile Compounds

Volatile compounds were isolated by headspace solid-phase microextraction (HS-SPME, 50/30 μm DVB/Car/PDMS fiber-Supelco Inc., Bellefonte, PA, USA) and analyzed using gas chromatography/mass spectrometry detection (GC-MS). Before analysis, each sample (3 g) was transferred into a 22 mL vial, combined with 400 µL of 2-pentanol-4-methyl (9.8 mg/L), used as the internal standard, and closed with a screw cap equipped with a Teflon-lined silicone rubber septum (Supelco Inc., Bellefonte, PA, USA). Before the first extraction, the fiber was conditioned in the GC injector port at 250 °C for 1 h, according to the manufacturer’s recommendation. The extraction temperature of the headspace was 40 °C for 10 min. The samples were mildly vortexed during extraction using a magnetic stirrer. Fiber exposition was prolonged for 30 min at 40 °C. Thermal desorption in the splitless mode was performed in the injector at 250 °C for 2 min into a Finnegan Trace MS for GC/MS (Agilent 6890 Series GC system, Agilent 5973 Net Work Mass Selective Detector; Milan, Italy) equipped with a DB-WAX capillary column (Agilent Technologies; 30 m, 0.250 mm inner diameter, film thickness 0.25 μm). The GC–MS system and chromatographic conditions described by Corona et al. [12] were used for analysis. Volatile organic compounds were identified through a comparison of the mass spectra and GC retention times with those of the pure commercial standard compounds and by comparing their mass spectra with those within the NIST/EPA/NIH Mass Spectral Library database (Version 2.0d, build 2005). For volatile organic compounds without the commercially available standard, their identification was conducted by matching their mass spectrum with those of the NIST library or reported in the literature. The quantification of the compounds identified was expressed in relation to the internal standard used. The determinations were carried out on three different samples.

### 2.6. Statistical Analysis

Data are reported as means ± standard deviation and are the results of the analysis of three replicates of each sample. Analysis of variance (ANOVA) and Tukey’s multiple range tests with *p* ≤ 0.05 were conducted with the SPSS Version 20.0 statistical software package (SPSS Inc., Chicago, IL, USA). All physical and chemical analyses reported above were carried out on three different samples.

## 3. Results and Discussion

### 3.1. Physicochemical Characterization

For the physicochemical characterization of the anchovy sauces’ analyses of pH, moisture content (%), and color were carried out. Table 2 shows that the pH values of the commercial and experimental samples were weakly acidic, varying between 5.41 and 5.84, which was in agreement with the guidelines of PDO [7] and with other authors [13,14]. This acidic character was due to the fermentation and dissociation of amino acids and small peptides in the presence of a high salt concentration [14] (Table 2). 

The moisture content of the experimental samples decreased with increasing maturation time (Table 2) (R3 < R2 < R1), while the moisture of the commercial samples, of which the ripening time was unknown, differed and was higher than that of R3. As reported in the disciplinary, sauce maturation occurs in wooden vats that are not hermetically sealed and can allow partial water evaporation, causing sauce concentration. The values of water activity of all the samples ranged from 0.75 to 0.78.

Color is an important attribute of anchovy sauce, and the results of the color analysis are shown in Table 2. The values of L* ranged from 31.3 at 12 months (R1) to 25.6 at 48 months of maturation (R3) and, together with the redness index, a*, and the yellowness index, b*, showed that R3 was darker compared to the others. In practice, after 12 months of maturation (R1), the sample showed a color with a yellow/amber tone, changing to a darker color liquid after 48 months (R3). As can be seen—in particular, from the L* parameter, which decreased, indicating the sample browning—the effect of sample concentration (R3) also reflected on color changes, in agreement with what was observed for the moisture content. On the other hand, the darkening of the color can also be attributed to the formation of free amino acids and low-molecular-weight nitrogen compounds, as reported by Klomklao et al. [15]. The commercial samples also differed from each other and the R series samples, in terms of color characteristics. 

### 3.2. Free Amino Acids 

Relevant concentrations of essential and nonessential amino acids were found in the tested fish sauces, as shown in Table 3. In certain cultures, fish sauce has important nutritional value, as it provides a useful source of amino acids to humans [16]. It has been estimated that consumption of 15–30 mL of fish sauce per day could ensure protein intake, and thus provide essential amino acids. In fact, fish sauce could contain about 10 g/100 mL of amino acids, of which about 70% are free-form [5]. Together with other active flavor components, such as volatile fatty acids and ketones, free amino acids can influence flavor characteristics and help refine the aroma and taste of the sauce. In fact, some of them can help create sweetness, bitterness, sourness, and umami. It is well known that high levels of glutamate and aspartate produce umami flavor [17,18] and this justifies the fact that fish sauce is often used as a seasoning to add umami flavor and salt to foods. Glutamate was the dominant free amino acid in the tested fish sauce. It was present at concentrations ranging from 0.56 to 0.73 g/100 mL and, together with aspartate, showed a significant increase during maturation from 12 (R1) to 48 months (R3), indicating an increase in the concentration of substances responsible for the umami taste.

Along with glutamate and aspartate, the concentration of lysine, threonine, glycine, valine, serine, and proline increased significantly during maturation, especially between 24 and 48 months, while the concentration of leucine decreased within the 48 months of maturation.

In the commercial samples, similar to the R sample series, the most abundant amino acids were glutamate, aspartate, lysine, alanine, histidine, and histamine.

In addition, when looking at the total amount of amino acids, there was an increase during maturation (from R1 to R3) due to the proteolysis progress, which caused the free amino acids’ release, with values similar to those of the commercial samples. The total amount of amino acids, however, does not depend only on the ripening time but is also influenced by the quality of the raw material and many other environmental factors, such as temperature, humidity, etc.

### 3.3. Histamine

Histamine is one of the biogenic amines, basic nitrogen compounds formed mainly by the bacterial decarboxylation activity of free histidine in food [19,20]. Histamine can have a harmful effect on the health of the consumer. For this reason, the previously mentioned limits for fish products and fish sauce produced by fermentation have been established [10]. The decarboxylation activity of histidine is due to the action of histamine-producing halophilic bacteria, with the activity being highest at the beginning of stationary growth and in subsequent phases. As the stationary phase progresses, there is a decrease in bacterial activity. Histamine content in the sample is, therefore, related to both bacterial production and degradation activity [21], which is due to either histamine dehydrogenase or histamine oxidase, both of which are present in many bacterial species [22,23]. 

**Figure 1:**
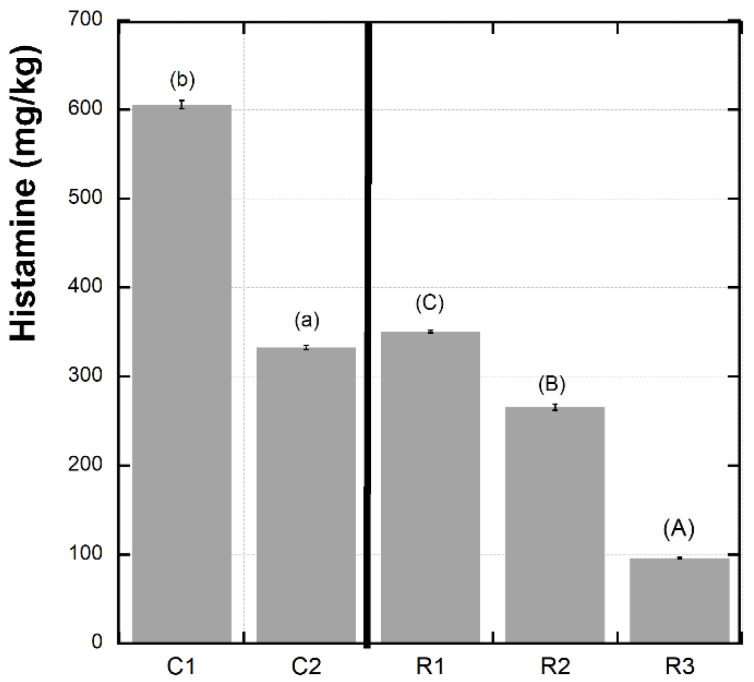
Histamine content in different anchovy sauce samples. Different letters between means indicate statistically significant differences at *p* < 0.05. Lower-case letters are used for comparing C1–C2 (commercial samples); upper-case letters are used for R1–R2–R3 samples at 12-, 24-, and 48-month ripening).

As shown in Figure 1, histamine content decreased significantly after 24 months (265.5 mg/kg) and decreased further after 48 months of maturation (96.0 mg/kg). This point is of particular interest, as it confirms the effectiveness of the PDO production specification in terms of fish sauce safety since it requires a fermentation period of at least 9 months before the sauce is placed on the market. Indeed, after 12 months, the histamine content (350 mg/kg) is close to the legal limits (400 mg/kg), and after 24 months, it drops to much lower levels (R2) and further decreases after 48 months (R3). It should be noted that one of the commercial products studied (C1) exceeded the legal limit for histamine. It is also worth noting that this sample had a different origin (Sicily region) and was not realized according to a disciplinary of production. Although the relationship between the decrease in histidine and the increase in histamine is not supported by the data obtained in the study, it could be that the histidine was microbially decarboxylated to form histamine. On the other hand, our results were consistent with the reports of Zaman et al. [24] on the existence of halophilic bacteria that degrade histamine in salted and fermented anchovy sauces. Such conclusions were also reported by Kuda et al. [25], who showed that bacteria can play the role of histamine suppressors during the long fermentation period of about 6 months to 2 years. This fact could explain the very low histamine content in long-fermented fish sauces [21]. It should be thought that, in the C1, the decarboxylation activity of histidine was the principal microbial activity, probably because the liquid (colatura) was spilled from the barrels after a brief period of ripening that did not allow the halophilic bacteria to degrade histamine through the activity of histamine dehydrogenase and/or histamine oxidase.

### 3.4. Volatile Compounds 

Several volatile substances were identified in the anchovy sauce samples (Table 4), including esters, alcohols, aldehydes, ketonic acids, and small amounts of nitrogen- and sulfur-containing compounds. The metabolism of the microorganisms involves the hydrolysis and lipolysis of proteins and fats, produced during the ripening of the chemical compounds responsible for the sensory properties of the fish sauce [26]. 

During ripening (R samples), the content of esters and the levels of alcohols and acids increased, while the concentration of aldehydes and ketones decreased. Branched amino acids (leucine, isoleucine, and valine) are degraded to their corresponding alcohols, acids, and aldehydes. Esters are formed through microbial metabolism, mainly from alcohols and carboxylic acids. After 48 months of aging, ethyl octanoate, ethyl nonanoate, ethyl phenyl acetate, and valerolactone are the main esters in fish sauce. All of these esters are responsible for the sweet smell reminiscent of fresh fruit, flowers, and honey. The content of esters in commercial samples varied widely. 

For some of the identified compounds, the sensory threshold value was taken from the literature and reported along with the odor activity value (OVA), which was calculated as the ratio between the concentration in the samples and the sensory threshold value.

Six alcohols contributed to the characteristic aroma of fish sauce in all samples, the three dominant ones being 1-heptanol, octanol, and phenethyl alcohol, with the latter having a floral, sweet, and honey-like note and being considered the main flavor component of most fermented foods (Table 5). As aging progressed up to 48 months, the alcohol content increased significantly, which may indicate that prolonged fermentation could improve the overall quality of the fish sauce. 

Aldehydes are important aromatic compounds in fermented fish sauce due to their low odor threshold; they are formed through the oxidative degradation of unsaturated fatty acids during fermentation [27]. Nonanal and octanal are oxidation products of oleic acid and are responsible for the green and fatty aroma [28]; their content decreased during maturation. Benzaldehyde is derived from phenylalanine and is often found in fish products that produce a pleasant almond aroma. Acetophenone was the most important of the volatile ketones, a product of lipid and/or amino acid degradation, with a sensory note characterized by green. Esters are present in most fermented seafood [29]; although their importance in the odor of fish sauce has not been fully elucidated, they contribute little to the odor of fish sauce due to their low concentrations and high odor thresholds. Pyridine is among the nitrogenous compounds that may be the source of the burnt and meat-like odor complexes in fish sauce.

Nineteen acidic compounds were detected (Table 4), most of which had high thresholds and, therefore, contributed little to the flavor [4,30]. The volatile acids detected in the fish sauce were mainly short- and medium-chain acids; the total amount of acidic compounds increased significantly from 60% of the total volatiles at 12 months of aging (R1) to over 85% at 48 months of aging (R3). The most important fatty acid was isovaleric acid, whose odor is associated with the smell of “cheddar cheese” [31]. In fermented fish sauce, alcoholic compounds originate mainly from the oxidative degradation of polyunsaturated fatty acids [4]. 

**Table 5:** Sensory threshold, descriptors, and odor activity value (OAV) of volatile compounds in different anchovy sauces.

Aroma Compounds	Sensory Threshold (ppb)	Descriptor	OAV				
			C1	C2	R1	R2	R3
**Alcohol**							
1-Hexanol	9	Herbaceous, grass [32]	2.80	1.62	2.26	2.43	5.63
1-Heptanol	2.4	Resin, floral, green [33]	167.85	103.64	94.04	99.06	102.94
1-Nonanol	45.5	Dusty, oily [30]	1.47	0.41	n.c.	n.c.	n.c.
1-Dodecanol	1.50	Floral, fruity, fatty [34]	18.55	113.52	37.01	31.75	28.60
**Aldehydes**							
1-Octanal	0.70	Honey, green [35]	89.35	311.17	218.22	n.c.	n.c.
Nonanal	1	Fatty, wax, soapy [36]	352.72	269.01	1022.16	1016.34	56.75
Decanal	6	Fruity, orange [33]	30.63	36.69	3.28	3.29	10.77
Phenylethanal	5	Hawthorne, honey [37]	9.45	5.28	2.60	2.33	n.c.
**Acids**							
Butanoic acid	240	Cheesy [38]	0.65	0.80	0.18	0.22	0.28
Isovaleric acid	30	Cheesy [39]	6.86	78.02	8.62	21.56	186.21
**Ketones**		Soap, green [1]					
Acetophenone	65		1.33	0.71	2.54	1.53	1.22
**others**							
Dimethyl trisulphide	0.36	Sulfur, rotten cabbage [40]	64.07	76.17	n.c.	n.c.	n.c.

C1–C2: Commercial samples. R1–R2–R3: sauce samples of the same producer at 12-, 24-, and 48-month ripening. n.c. = not calculable.

## 4. Conclusions

The present paper evaluated the changes in nutritional and volatile component characteristics for a typical anchovy sauce, colatura di alici di Cetara (PDO), during maturation in wooden vats within 48 months. The extension of the ripening period from 24 to 48 months allowed for a significant increase in the concentration of essential amino acids that contributed to improving the sauce’s nutritional value, while the histamine content decreased by about 27% from 12 to 48 months of ripening, thus improving product safety. As for the volatile compounds, during ripening, an overall increase in the aroma compounds was detected—in particular, the content of esters, alcohols, and acids increased—while the concentration of aldehydes and ketones decreased. The differences in composition and odor activity value between the commercial and PDO samples indicated differences in the manufacturing process, which, in the case of the PDO samples, is carried out according to ancient artisanal tradition. In summary, the longer maturation time results in an increase in nutritional properties, olfactory characteristics, and color, as well as making the fish sauce safer, as evidenced by a reduction in histamine. Overall, the information obtained in this experimentation contributes to the knowledge of this valuable product and can help to make the colatura of alici sauce known beyond the Italian borders.

## Figures and Tables

**Table 1:** Mobile phase and time/potential waveform used for amino acids analysis.

Mobile Phase (0.250 mL/min)				Time/Potential Waveform		
Time (min)	H_2_O (%)	NaOH (%)	NaOAc (%)	Time (sec)	Potential (V)	Integration
0.0	80	20	0	0.00	+0.13	
2.0	80	20	0	0.04	+0.13	
12.0	80	20	0	0.05	+0.28	
16.0	68	32	0	0.11	+0.28	began
24.0	36	24	40	0.12	+0.60	
40.0	36	24	40	0.41	+0.60	
40.1	20	80	0	0.42	+0.28	
42.1	20	80	0	0.56	+0.28	end
42.2	80	20	0	0.57	−1.67	
62.0	80	20	0	0.58	−1.67	
				0.59	+0.93	
				0.60	+0.13	

**Table 2:** Chemical–physical parameters determined in different anchovies sauces’ samples. Different superscript letters between means within a row indicate statistically significant differences at *p* < 0.05. Lower-case letters are used for comparing C1–C2 (commercial samples); upper-case letters are used for R1–R2–R3 samples at 12-, 24-, and 48-month ripening.

Samples	C1	C2	R1	R2	R3
moisture %	62.72 ± 0.47 ^a^	66.36 ± 0.50 ^b^	67.34 ± 0.40 ^C^	66.14 ± 0.50 ^B^	59.70 ± 0.45 ^A^
pH	5.41 ± 0.04 ^a^	5.81 ± 0.04 ^b^	5.73 ± 0.05 ^B^	5.54 ± 0.04 ^A^	5.84 ± 0.04 ^B^
L*	30.40 ± 0.23 ^b^	29.11 ± 0.22 ^a^	31.16 ± 0.62 ^B^	34.08 ± 0.26 ^C^	25.59 ± 0.20 ^A^
a*	12.74 ±0.10 ^b^	9.77 ± 0.08 ^a^	11.21 ± 0.11 ^B^	11.32 ± 0.09 ^B^	2.72 ± 0.02 ^A^
b*	7.61 ± 0.06 ^b^	5.50 ± 0.04 ^a^	11.73 ± 0.06 ^B^	13.63 ± 0.10 ^C^	0.23 ± 0.00 ^A^
C	14.84 ± 0.12 ^b^	11.21 ± 0.09 ^a^	16.22 ± 0.12 ^B^	17.74 ± 0.14 ^C^	2.73 ± 0.11 ^A^
Hue	30.85 ± 0.00 ^b^	29.37 ± 0.02 ^a^	46.30 ± 0.16 ^B^	50.21 ± 0.01 ^C^	4.82 ± 0.03 ^A^

**Table 3:** Free amino acids’ contents in different fish sauces (g/100 mL). Different superscript letters between means within a row indicate statistically significant differences at *p* < 0.05. Lower-case letters are used for comparing C1–C2 (commercial samples); upper-case letters are used for R1–R2–R3 samples at 12-, 24-, and 48-month ripening.

	AMINO ACIDS	C1	C2	R1	R2	R3
Non ESSENTIAL	Arginine	0.167 ± 0.033 ^a^	0.182 ± 0.025 ^a^	0.162 ± 0.020 ^A^	0.154 ± 0.013 ^A^	0.151 ± 0.044 ^A^
	Hydroxylysine	0.210 ± 0.021 ^a^	0.270 ± 0.038 ^a^	0.206 ± 0.011 ^B^	0.115 ± 0.006 ^A^	0.201 ± 0.006 ^B^
	Glutamine	0.291 ± 0.002 ^b^	0.168 ± 0.017 ^a^	0.255 ± 0.003 ^B^	0.275 ± 0.002 ^C^	0.227 ± 0.011 ^A^
	Alanine	0.597 ± 0.013 ^b^	0.437 ± 0.037 ^a^	0.403 ± 0.012 ^A^	0.639 ± 0.041 ^C^	0.568 ± 0.001 ^B^
	Glycine	0.275 ± 0.136 ^a^	0.227 ± 0.009 ^a^	0.248 ± 0.004 ^A^	0.249 ± 0.026 ^A^	0.407 ± 0.006 ^B^
	Serine	0.369 ± 0.032 ^a^	0.358 ± 0.001 ^a^	0.262 ± 0.021 ^A^	0.342 ± 0.008 ^B^	0.477 ± 0.013 ^C^
	Proline	0.214 ± 0.011 ^b^	0.182 ± 0.001 ^a^	0.189 ± 0.010 ^A^	0.189 ± 0.018 ^A^	0.361 ± 0.024 ^B^
	Glutamate	0.697 ± 0.081 ^a^	0.642 ± 0.017 ^a^	0.555 ± 0.026 ^A^	0.562 ± 0.022 ^A^	0.727 ± 0.018 ^B^
	Aspartate	0.611 ± 0.039 ^b^	0.504 ± 0.027 ^a^	0.447 ± 0.016 ^A^	0.438 ± 0.005 ^A^	0.608 ± 0.019 ^B^
	Tyrosine	0.021 ± 0.003 ^a^	0.063 ± 0.009 ^b^	0.051 ± 0.001 ^B^	0.060 ± 0.001 ^C^	0.036 ± 0.003 ^A^
ESSENTIAL	Lysine	0.572 ± 0.008 ^a^	0.546 ± 0.024 ^a^	0.415 ± 0.008 ^A^	0.419 ± 0.012 ^A^	0.543 ± 0.009 ^B^
	Threonine	0.374 ± 0.023 ^a^	0.374 ± 0.015 ^a^	0.312 ± 0.010 ^A^	0.378 ± 0.002 ^B^	0.539 ± 0.002 ^C^
	Valine	0.367 ± 0.018 ^b^	0.326 ± 0.011 ^a^	0.360 ± 0.018 ^A^	0.354 ± 0.032 ^A^	0.510 ± 0.013 ^B^
	Isoleucine	0.325 ± 0.029 ^b^	0.265 ± 0.018 ^a^	0.295 ± 0.011 ^A^	0.246 ± 0.040 ^A^	0.256 ± 0.009 ^A^
	Leucine	0.423 ± 0.047 ^a^	0.371 ± 0.041 ^a^	0.467 ± 0.036 ^B^	0.455 ± 0.028 ^B^	0.285 ± 0.014 ^A^
	Methionine	0.286 ± 0.035 ^b^	0.196 ± 0.017 ^a^	0.154 ± 0.021 ^A^	0.158 ± 0.015 ^A^	0.153 ± 0.005 ^A^
	Cysteine	0.041 ± 0.009 ^a^	0.040 ± 0.001 ^a^	0.036 ± 0.001 ^B^	0.032 ± 0.001 ^A^	0.044 ± 0.001 ^C^
	Histidine	0.406 ± 0.034 ^a^	0.428 ± 0.010 ^a^	0.345 ± 0.020 ^B^	0.288 ± 0.017 ^A^	0.383 ± 0.024 ^B^
	Phenylalanine	0.298 ± 0.020 ^b^	0.174 ± 0.020 ^a^	0.217 ± 0.017 ^A^	0.209 ± 0.009 ^A^	0.265 ± 0.007 ^B^
	**TOTAL**	**6.54 ± 0.59 ^a^**	**5.75 ± 0.34 ^a^**	**5.40 ± 0.26 ^A^**	**5.56 ± 0.30 ^A^**	**6.74 ± 0.23 ^B^**

C1–C2: Commercial samples. R1–R2–R3: sauce samples of the same producer at 12-, 24-, and 48-month ripening.

**Table 4:** Concentration (μg/L) of volatile compounds identified in colatura di alici samples. Different superscript letters between means within a row indicate statistically significant differences at *p* < 0.05. Lower-case letters are used for comparing C1–C2 (commercial samples); upper-case letters are used for R1–R2–R3 samples at 12-, 24-, and 48-month ripening.

	C1	C2	R1	R2	R3
Ethyl octanoate	64.65 ± 1.91 ^b^	51.37 ± 1.52 ^a^	38.37 ± 0.98 ^A^	39.89 ± 1.18 ^A^	56.20 ± 1.66 ^B^
Ethyl nonanoate	25.79 ± 0.74 ^a^	57.91 ± 1.65 ^b^	18.22 ± 0.56 ^A^	17.75 ± 0.51 ^A^	53.54 ± 1.53 ^B^
Butyryl lactone	62.90 ± 1.59 ^b^	49.63 ± 1.25 ^a^	22.23 ± 0.66 ^A^	28.44 ± 0.72 ^cC^	24.06 ± 0.61 ^B^
Ethyl phenylacetate	n.d.	19.41 ± 0.93	n.d.	13.25 ± 0.51 ^aA^	165.15 ± 7.04 ^B^
-Valerolactone	61.75 ± 2.95 ^a^	104.53 ± 4.99 ^b^	32.55 ± 1.67 ^A^	34.94 ± 1.67 ^A^	71.07 ± 3.39 ^B^
Ethyldodecanoate	5.97 ± 0.28	n.d.	9.27 ± 0.22 ^B^	7.58 ± 0.29 ^A^	12.64 ± 0.54 ^C^
**Total Esters**	**221.07 ± 7.46 ^a^**	**282.85 ± 10.33 ^b^**	**120.64 ± 4.09 ^A^**	**141.86 ± 4.87 ^B^**	**382.66 ± 14.76 ^C^**
1-Hexanol	25.17 ± 0.82 ^b^	14.62 ± 0.48 ^a^	20.33 ± 0.62 ^A^	21.84 ± 0.71 ^A^	50.64 ± 1.65 ^B^
1-Heptanol	402.83 ± 9.18 ^b^	248.73 ± 5.67 ^a^	225.70 ± 3.27 ^A^	237.75 ± 5.42 ^B^	247.05 ± 5.63 ^B^
2-Ethyl-Hexanol	49.26 ± 1.10 ^a^	66.27 ± 1.48 ^b^	198.18 ± 6.12 ^C^	126.26 ± 2.82 ^B^	76.27 ± 1.70 ^A^
1-Octanol	34.29 ± 1.88 ^b^	11.56 ± 0.58 ^a^	96.05 ± 3.05 ^B^	95.45 ± 2.93 ^B^	81.50 ± 2.50 ^A^
1-Nonanol	67.00 ± 1.50 ^b^	18.50 ± 0.41 ^a^	n.d.	n.d.	n.d.
a-Terpineol (terpenoid)	21.84 ± 0.48	n.d.	n.d.	n.d.	n.d.
Phenylethylalcohol	12.92 ± 0.29 ^a^	250.06 ± 5.58 ^b^	31.78 ± 0.83 ^A^	57.95 ± 1.29 ^B^	211.92 ± 4.73 ^C^
1-Dodecanol	27.82 ± 1.03 ^a^	170.28 ± 6.29 ^b^	55.52 ± 4.22 ^B^	47.62 ± 1.76 ^A^	42.90 ± 1.59 ^A^
**Total Alcohols**	**641.14 ± 16.28 ^a^**	**780.02 ± 20.49 ^b^**	**627.56 ± 18.11 ^B^**	**586.88 ± 14.93 ^A^**	**710.27 ± 17.80 ^C^**
1-Octanal	62.54 ± 1.76 ^a^	217.82 ± 6.12 ^b^	152.76 ± 6.22	n.d.	n.d.
Nonanal	352.72 ± 11.20 ^b^	269.01 ± 8.54 ^a^	1022.16 ± 15.38 ^B^	1016.34 ± 32.28 ^B^	56.75 ± 1.80 ^A^
Decanal	183.76 ± 8.06 ^a^	220.12 ± 9.66 ^b^	19.71 ± 1.05 ^A^	19.74 ± 0.87 ^A^	64.63 ± 2.84 ^B^
Benzaldehyde	84.19 ± 2.38 ^a^	123.15 ± 3.48 ^b^	76.95 ± 3.55 ^A^	114.16 ± 3.22 ^B^	288.39 ± 8.14 ^C^
Phenylethanal	47.27 ± 1.06 ^b^	26.39 ± 0.59 ^a^	12.99 ± 0.78 ^B^	11.63 ± 0.26 ^A^	n.d.
2-Undecenal	11.99 ± 0.52	n.d.	98.74 ± 3.15 ^B^	36.55 ± 1.40 ^A^	n.d.
**Total Aldehydes**	**742.47 ± 24.97 ^a^**	**856.49 ± 28.39 ^b^**	**1383.31 ± 30.13 ^C^**	**1198.43 ± 38.03 ^B^**	**409.77 ± 12.78 ^A^**
Acetic acid	1040.52 ± 38.73 ^b^	660.35 ± 24.58 ^a^	66.29 ± 1.99 ^A^	77.28 ± 2.88 ^A^	517.09 ± 19.25 ^B^
Propionic acid	61.66 ± 1.95 ^a^	157.16 ± 4.96 ^b^	13.88 ± 0.77 ^A^	15.29 ± 0.48 ^A^	61.49 ± 1.94 ^B^
Isobutyric acid	214.89 ± 8.10 ^a^	314.22 ± 11.83 ^b^	29.56 ± 0.15 ^A^	37.82 ± 1.42 ^A^	439.05 ± 16.54 ^B^
Butanoic acid	157.10 ± 5.81 ^a^	193.14 ± 7.14 ^b^	49.27 ± 2.11 ^A^	53.96 ± 1.99 ^A^	67.77 ± 2.50 ^B^
Isovaleric acid	205.95 ± 7.98 ^a^	2340.67 ± 90.65 ^b^	258.67 ± 5.24 ^A^	646.82 ± 25.05 ^B^	5586.36 ± 216.35 ^C^
Pentanoic acid	27.76 ± 1.20 ^a^	60.85 ± 2.63 ^b^	24.58 ± 0.44 ^A^	27.58 ± 1.19 ^B^	35.63 ± 1.54 ^C^
Hexanoic acid	85.91 ± 3.17 ^a^	351.91 ± 12.99 ^b^	77.25 ± 3.18 ^A^	84.50 ± 3.12 ^A^	276.22 ± 10.10 ^B^
Eptanoic acid	27.82 ± 1.06 ^a^	63.39 ± 2.43 ^b^	58.86 ± 1.58 ^B^	62.64 ± 2.40 ^B^	31.05 ± 1.19 ^A^
Octanoic acid	62.90 ± 1.90 ^a^	158.84 ± 4.81 ^b^	69.64 ± 1.11 ^A^	71.60 ± 2.17 ^A^	76.34 ± 2.31 ^B^
Nonanoic acid	65.46 ± 2.25 ^a^	154.61 ± 5.32 ^b^	408.52 ± 9.66 ^B^	489.74 ± 16.85 ^C^	116.00 ± 3.99 ^A^
Decanoic acid	108.52 ± 4.46 ^a^	184.27 ± 7.58 ^b^	130.83 ± 4.33 ^B^	123.74 ± 5.09 ^B^	104.32 ± 4.29 ^A^
Benzoic acid	136.86 ± 5.81 ^a^	229.96 ± 9.76 ^b^	56.87 ± 2.81 ^A^	68.06 ± 2.89 ^A^	407.59 ± 17.29 ^B^
Dodecanoic acid	107.20 ± 3.67 ^a^	232.96 ± 7.98 ^b^	136.55 ± 4.21 ^C^	93.61 ± 3.20 ^B^	73.84 ± 2.53 ^A^
Phenylacetic acid	n.d.	26.80 ±1.03 ^a^	n.d.	n.d.	516.09 ± 21.99
Tetradecanoic acid	383.27 ± 18.11 ^a^	821.60 ± 38.82 ^b^	589.10 ± 18.22 ^C^	332.15 ± 15.69 ^B^	288.16 ± 13.61 ^A^
Pentadecanoic acid	65.78 ± 2.17 ^a^	133.14 ± 4.40 ^b^	68.78 ± 3.25 ^C^	62.15 ± 2.05 ^B^	42.85 ± 1.42 ^A^
Hexadecanoic acid	1912.37 ± 72.37 ^a^	2727.94 ± 103.23 ^b^	1478.50 ± 34.68 ^C^	1066.51 ± 40.36 ^B^	797.53 ± 30.18 ^A^
Heptadecanoic acid	56.41 ± 2.15 ^a^	89.03 ± 3.40 ^b^	49.33 ± 0.25 ^B^	53.08 ± 2.03 ^C^	16.34 ± 0.62 ^A^
Octadecanoic acid	567.29 ± 25.96 ^a^	837.46 ± 38.32 ^b^	352.88 ± 13.13 ^B^	347.01 ± 15.88 ^B^	230.32 ± 10.54 ^A^
**Total Acids**	**5287.66 ± 206.84 ^a^**	**9738.36 ± 381.84 ^b^**	**3919.36 ± 107.11 ^A^**	**3713.55 ± 144.74 ^A^**	**9684.03 ± 378.28 ^B^**
Acetophenone	86.71 ± 1.94 ^b^	46.20 ± 1.03 ^a^	165.42 ± 4.02 ^C^	99.65 ± 2.22 ^B^	79.11 ± 1.77 ^A^
1,2-Cyclopentanedione	52.66 ± 1.18 ^b^	4.29 ± 0.10 ^a^	n.d.	12.58 ± 0.28	n.d.
2-Pentadecanone	30.82 ± 1.06 ^a^	67.15 ± 2.31 ^b^	18.16 ± 0.34 ^B^	21.23 ± 0.73 ^C^	15.70 ± 0.54 ^A^
**Total Ketones**	**170.19 ± 4.17 ^b^**	**117.64 ± 3.43 ^a^**	**183.58 ± 4.36 ^C^**	**133.45 ± 3.23 ^B^**	**94.81 ± 2.30 ^A^**
Pyridine	133.77 ± 2.99 ^a^	176.15 ± 3.93 ^b^	182.11 ± 1.40 ^A^	187.35 ± 4.18 ^B^	236.05 ± 5.27 ^C^
**Nitrogen-containing**					
Dimethyl trisulfide	23.07 ± 0.86 ^a^	27.42 ± 1.03 ^b^	n.d.	n.d.	n.d.
**Sulfur-containing**					

n.d., not detected.

## Data Availability

The data presented in this study are available on request from the corresponding author.
